# Kupffer Cells Mediate Systemic Antifungal Immunity

**DOI:** 10.1016/j.it.2019.11.001

**Published:** 2019-12

**Authors:** Carolina Coelho, Rebecca A. Drummond

**Affiliations:** 1 Medical Research Council Centre for Medical Mycology, Department of Biosciences, College of Life and Environmental Sciences, University of Exeter, Exeter, UK; 2 Institute of Immunology and Immunotherapy, Institute of Microbiology and Infection, University of Birmingham, Birmingham, UK

## Abstract

Patients with liver dysfunction have increased susceptibility to fungal infections. A recently published article (Sun *et al.*) describes the potential mechanism underlying this association, which maps to the antifungal activity of liver-resident Kupffer cells. This research highlights the importance of understanding tissue-specific immune responses in disease pathogenesis.

Kupffer cells are an abundant population of liver-resident macrophages that play key roles in nearly all aspects of liver homeostasis and disease [1]. In recent years there has been significant progress in understanding the identity and functions of these specialized macrophages, including their role as a key immune barrier by clearing bacteria and viruses from the bloodstream. Using a combination of intravital microscopy and macrophage-depletion models, Sun and colleagues [2] now show that Kupffer cells are crucial for clearing systemic fungal infection. The authors focus on fungal infection caused by *Cryptococcus neoformans* which kills ∼200 000 people each year. Liver dysfunction, particularly caused by cirrhosis and/or chronic hepatitis, is an independent risk factor for developing cryptococcosis and other fungal diseases [3], but the specific mechanisms connecting liver dysfunction to lethal fungal infection have remained unclear.

Using intravital microscopy in mice, the authors visualized Kupffer cells interacting with and ingesting *C. neoformans* yeast within 1 h following intravenous infection [2]. To demonstrate the contribution of these interactions to pathogenesis, the authors depleted Kupffer cells using either clodronate liposomes or gadolinium chloride: Kupffer cells were found to filter fungal organisms from the bloodstream, and this reduced fungal dissemination to other organs. Using a series of gene-deficient mice (*C3*^−/−^*, CRIg*^−/−^*, C5*^−/−^), the authors demonstrated that the Kupffer cell–yeast interactions were dependent on complement protein C3, its receptor CRIg, and to a lesser extent C5 (Figure 1). The minor role of C5 was surprising because C5 deficiency significantly enhances susceptibility to cryptococcosis in mice [4]. This study therefore highlights the poorly understood role of complement in systemic fungal infection, and suggests that it may function in an organ-specific fashion.

**Figure 1:**
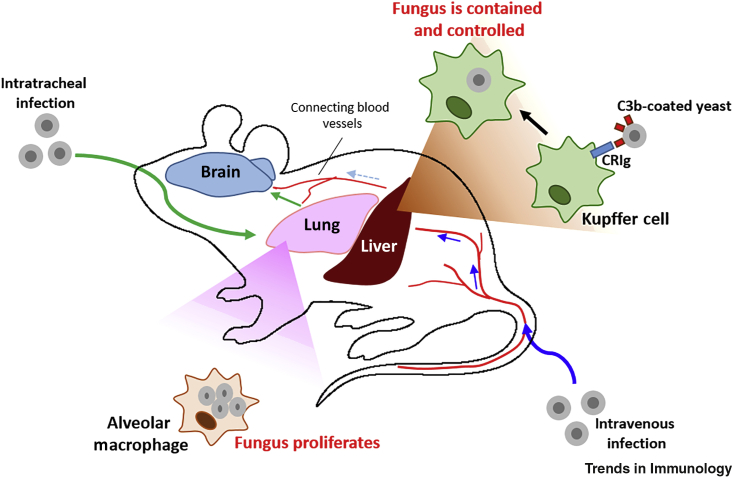
Fungal Pathogen Dissemination and Organ Burden Are Influenced by Tissue-Resident Macrophages. Sun *et al.* [2] show that intravenous infection (blue arrows) with *Cryptococcus neoformans* in mice results in the capture of circulating yeast cells by Kupffer cells with the aid of the complement C3–CRIg pathway. This capture clears fungus from the bloodstream and decreases dissemination to the lung and brain. When *C. neoformans* is inoculated directly into the lung (green arrows), yeast cells proliferate within alveolar macrophages and allow the establishment of infection in this organ, accompanied by fungal dissemination to other tissue sites.

Moreover, this study is a remarkable illustration of how different tissue-resident macrophages respond to an infectious challenge, and how these responses affect organ susceptibility. Although Kupffer cells seemed to be unable to completely kill ingested yeast, they inhibited yeast growth for several days while ingestion by alveolar macrophages in the lung resulted in quick proliferation of yeast cells. Growth inhibition within Kupffer cells was independent of interferon (IFN)-γ–IFNR1 signaling because *Ifnr1*^−/−^ animals showed no defects in Kupffer cell fungal capture and control, in line with other work demonstrating that Kupffer cells do not require IFN-γ for their antibacterial activity [1]. This is an important conundrum because IFN-γ is considered to be the most important cytokine for anticryptococcal protection [5]. Thus, it will be important to delineate the protective actions of IFN-γ in a cell- and timing-specific manner, especially because IFN-γ-based therapies are currently being trialed in the clinic for treatment of cryptococcal meningitis.

By examining liver-specific pathways of immune protection, Sun and colleagues have made important mechanistic insights into a poorly understood risk factor for invasive fungal infections. Similarly, we recently showed that brain-resident macrophages (i.e., microglia) efficiently recognized fungal toxins and established a CARD9-dependent proinflammatory pathway to recruit neutrophils to fungus-infected brains in both mice and humans [6]; these findings contribute to explaining why patients with *CARD9* mutations show a striking susceptibility to brain fungal infections. More studies such as these will be necessary to determine the pathways of immune protection operating at different tissue sites, and this is becoming increasingly possible given recent advances in our understanding of tissue-resident macrophage lineages and maintenance. For example, ZEB2 was recently identified as a master transcription factor in mice that maintains tissue-resident macrophage identity, including Kupffer cells and lung alveolar macrophages [7]; this in turn led to the development of new fate-mapping tools (e.g., *Clec4f–cre × Zeb2*^fl/fl^ transgenic mice) to enable the specific manipulation of tissue-resident macrophages [7]. These tools will be crucial for the dissection of organ-specific immunity in future studies.

*C. neoformans* exhibits strong tropism for the brain, and exhibits a high capacity for invading the brain vasculature. The mechanisms underlying this are poorly understood, but one hypothesis (the Trojan horse) posits that yeast cells, when engulfed by macrophages or monocytes, can be carried by immune cells to distant organs such as the brain. However, Sun *et al.* demonstrate that, in the first hours after infection, circulating monocytes do not contribute to clearing yeast from the bloodstream or preventing its dissemination to the brain [8]; indeed, relative to controls, depletion of either inflammatory monocytes (using *Ccr2*^−/−^ mice) or patrolling monocytes (using *Nur77*^−/−^ mice) still resulted in efficient killing of yeast cells within the liver and their subsequent clearance from the bloodstream. By contrast, older studies using a clodronate-based depletion strategy similar to that used by Sun *et al.* reported converse data: when mice were intravenously infected with *C. neoformans* and treated with clodronate liposomes 3 days after infection, the fungal burden in the brain was decreased compared with nonclodronate-depleted mice [8]. Clearly warranting further investigation, these discrepancies might be driven by differing degrees of inflammation caused by the different clodronate treatment protocols used. Regardless, these findings highlight a need for better tools to deplete and manipulate tissue-resident macrophages, such as in the case of ZEB2 transgenic mice, as outlined above.

Lastly, an exciting aspect of the study by Sun and coworkers is the observed transient fungemia in the *C. neoformans*-infected mice [2]. Although most experiments in this study used the intravenous route of infection, the authors also mimicked a natural infection route by depositing yeast directly into the lung with intratracheal inoculation. In this case they still observed a beneficial effect of Kupffer cells in decreasing brain fungal burden relative to controls. This suggests that there is a point during infection when yeast escape the lung and are exposed to Kupffer cell clearing. In line with this, we recently observed that *C. neoformans* can disseminate from mouse nares to the brain in as little as 3 h [9]*,* whereas others have shown that spores of C*. neoformans* are transported into mouse lymph nodes within the first 24 h after intranasal infection [10]. All these studies strongly suggest that brain dissemination occurs early in yeast infection in mice (and not as a consequence of lung infection). This in turn underscores the need for further detailed studies to understand different routes of microbial dissemination.

Collectively, we are now starting to unravel how pathogens disseminate through the host, and how tissue-resident macrophages skew the balance of infection in different organs (Figure 1). These mechanisms help us to understand organ-specific host–fungus interactions which may allow improvements in treatments for cryptococcosis and other invasive fungal infections that are urgently needed to reduce the global burden of these diseases.
